# Lateral Calcaneal Artery Perforator/Propeller Flap in the Reconstruction of Posterior Heel Soft Tissue Defects

**DOI:** 10.1055/s-0044-1791764

**Published:** 2024-10-24

**Authors:** T.M Balakrishnan, Liza J., Abinaya Karthikeyan, Srividhya Madhurbootheswaran, M. Sugumar, M. Sridharan

**Affiliations:** 1 Department of Plastic and Faciomaxillary Surgery, Madras Medical College, Chennai, Tamil Nadu, India

**Keywords:** perforator flaps, propeller flaps, lateral calcaneal artery, posterior heel defects

## Abstract

**Introduction** Posterior heel soft tissue defects with/without tendoachilles injury are difficult to reconstruct because of bony prominence, limited expendable local soft tissues, and significant donor site morbidity. In this study, we evaluate the lateral calcaneal artery (LCA) perforator/propeller flap for posterior heel soft tissue defect reconstruction.

**Materials and Methods** A preliminary cadaveric study was done on 22 specimens to study the course of the LCA and its perforators. The knowledge gleaned from the cadaveric study was applied in our clinical study. The clinical study was conducted from January 2018 to May 2023 in 13 patients (10 males and 3 females) with posterior heel defects. The primary movement of the perforator flaps were either V-Y advancement ( *n* = 10) or propeller ( *n* = 3).

**Results** All the flaps settled well except for superficial epidermolysis in one patient (7.69%) which healed conservatively. The average follow-up period was 14.53 months. Postoperatively, all patients had normal gait and normal range of movements. The LCA with the maximum number of perforators in the retrofibular/retromalleolar segment and the islanded flaps based on these perforators provided the excellent coverage for tendoachilles insertion defects and posterior heel defects facilitating excellent shoeability in all cases.

**Conclusion** The study concludes that the LCA perforator/propeller flap may be a reliable cover for the posterior heel defects with good color, texture, thickness, and contour match rendering a shoeable stable foot.

## Introduction

Reconstruction of soft tissue defects over the posterior heel with/without tendoachilles injury is still one of the challenging problems faced by the reconstructive surgeons. 1 2 It exacts the expertise of the reconstructive surgeon as it necessitates a sensate and thin flap. 1 2 The usual options available are transposition flaps, 3 reverse flow flaps, 4 and free flaps. 5 Transposition flap produces donor contour deformity. 3 Reverse superficial sural artery flap sometimes suffers from venous congestion and necrosis. 4 Free flap is technically demanding and labor intensive. 5 Perforator/propeller flaps 6 7 8 9 overcome all these shortcomings. These perforator propeller flaps provide like tissue with color, texture, and thickness match with supranormal homogenized blood supply and intact sensation. 6 Many times the donor site can be primarily closed, thereby minimizing the morbidity. 6 The classical pedicled lateral calcaneal artery (LCA) flap, first described by Grabb and Argenta in 1981, is undoubtedly one of the best methods for coverage of soft tissue defects of the ankle. 10 11 The pedicled LCA flap was used extensively as a local flap to cover the posterior heel defects but it caused contour deformity at the donor site and loss of sensation to the lateral border of the foot. 10 In this study, we introduce and analyze the outcomes of LCA perforator-based advancement/propeller flap for the reconstruction of posterior heel defects.

## Anatomical Study

### Materials and Methods

**Fig. 1:**
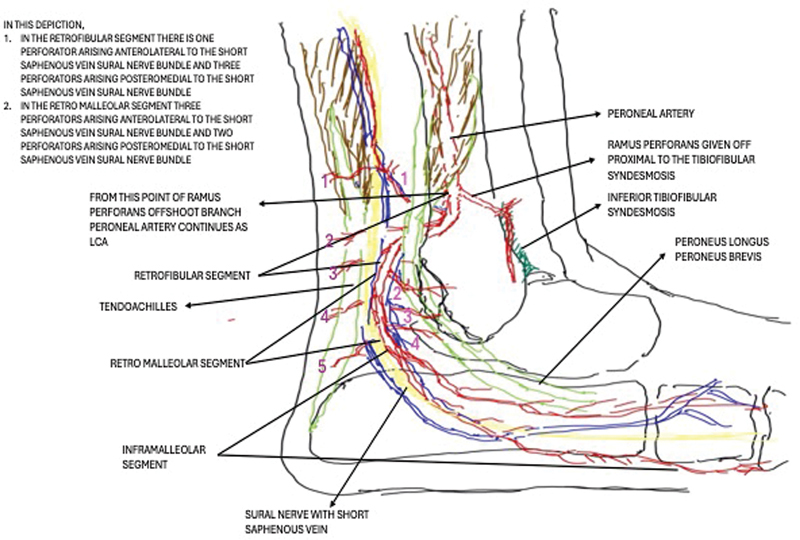
Schematic diagram depicting the various segments of the lateral calcaneal artery (LCA).

**Fig. 2:**
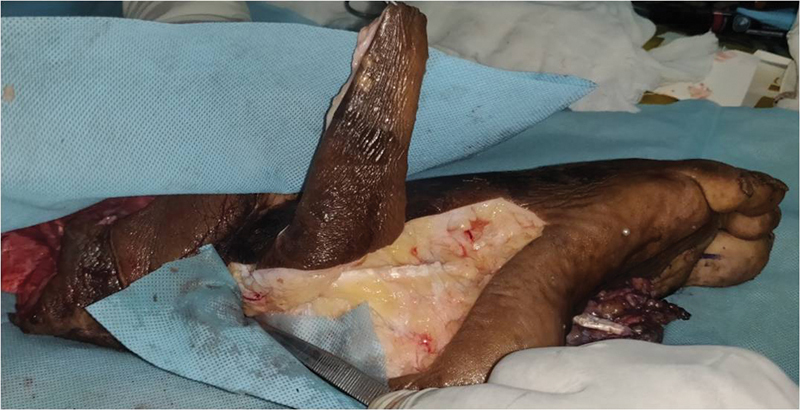
Incisions for anatomical dissection of lateral calcaneal artery (LCA).

A preliminary cadaveric study was done in 22 specimens to study the course of LCA segments and its perforators. The injection and dissection studies were conducted from June 2017 to January 2018. Twenty fresh cadaver legs and two amputation specimens were examined. After the injection of red lead oxide into the proximal popliteal artery and methylene blue into the proximal popliteal vein, the specimens were stored overnight in the freezer. An incision was placed over the tendoachilles starting from 10 cm above the tip of the lateral malleolus down to the lateral calcaneal aspect. The dissection was performed over the tenosynovium of tendoachilles into the deep fat pad to identify the two segments of LCA, which were situated posterior and deep to the small saphenous vein and sural nerve. By definition, LCA was the continuation of the peroneal artery after its ramus perforans branch. 12 In our study, we divided the LCA into two segments ( Fig. 1 ): (1) Retrofibular/retromalleolar segment (this segment ran from the upper border of lower tibiofibular syndesmosis as the continuation of peroneal artery beyond its ramus perforans branch. It continued in the deep cleft between the tendoachilles and peroneal tendons up to the tip of the lateral malleolus). (2) The inframalleolar segment (which was extending in a curved manner below the tip of the lateral malleolus to the base of the fifth metatarsal bone) ( Fig. 2 ). A parallel anterior incision was placed over the peroneal compartment and was extended over the lateral malleolus then horizontally extended up to the base of the fifth metatarsal bone ( Fig. 2 ). Dissection was performed to delineate the course of the LCA, branching pattern, and its perforators (location, size, distribution). In this study, the number, size, location, and course of perforators from the retrofibular/retromalleolar segment of the LCA were only studied. The calipers (with micrometer accuracy) and scale were used for measurements. The distal end of the peroneal artery was identified by dissecting through the beefy distal portion of the flexor hallucis longus. The point at which the ramus perforans was given was then identified. Then the dissection was performed along the LCA.

## Results

**Fig. 3:**
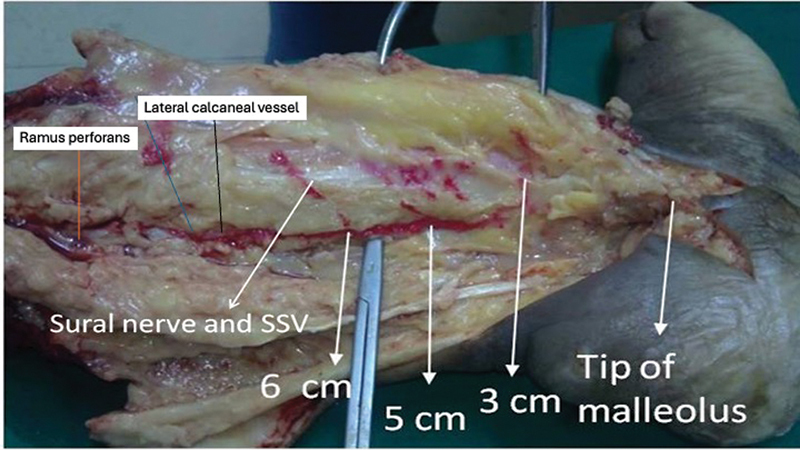
Cadaver dissection showing the distribution of perforators from the retrofibular/retromalleolar segment of the lateral calcaneal artery (LCA).

**Fig. 4:**
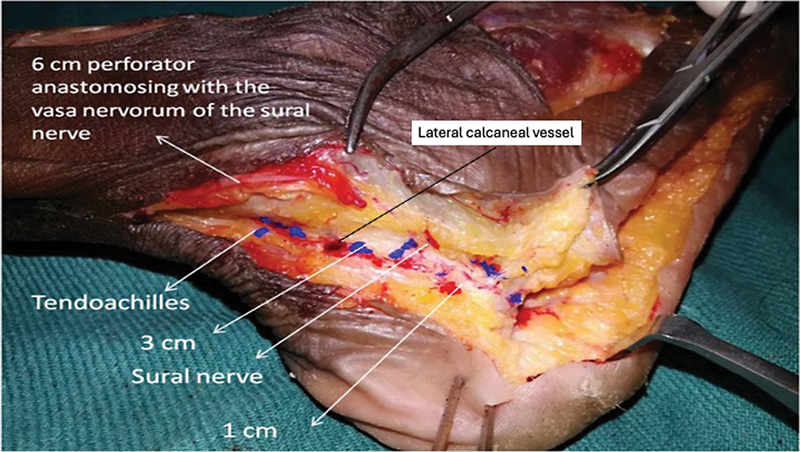
Anatomical injection study showing the distribution of perforators from the retrofibular/retromalleolar segment of the lateral calcaneal artery (LCA) in relation to small saphenous vein and sural nerve.

The average length (beyond ramus perforans to the tip of the lateral malleolus) of the retrofibular/retromalleolar segment of the LCA was 7.5 cm. The inframalleolar segment was divided into an average of 2.5 branches and was traceable up to an average distance of 1.25 cm from the base of the fifth metatarsal bone. Superiorly, the branches disappeared beneath the peroneus longus **/** brevis tendons to anastomose with the lateral tarsal artery and the descending branch of ramus perforans near the extensor digitorum brevis. The inferior branch disappeared beneath the abductor digiti minimi to anastomose with the lateral plantar artery perforators. Only submillimetric filiform perforators were encountered with the inframalleolar segment of LCA. There was 3.27 average number of perforators arising from the retrofibular/retromalleolar segment of LCA ( Table 1 ) ( Fig. 3 ). Most of the perforators from the LCA retrofibular/retromalleolar segment were located within 7.5 cm from the tip of the lateral malleolus (cranial direction) ( Fig. 1 ). The cranial-most perforator was just distal to the ramus perforans, which was approximately 7.5 cm above the lateral malleolar tip and the caudal-most perforator was approximately 1 cm above the tip ( Table 1 ). In three cases, the proximal-most perforator from this segment anastomosed with the median superficial sural artery—the vasa nervorum of the sural nerve before reaching the skin ( Fig. 4 ). In our study, 53 (73.61%) perforators were posteromedial to the small saphenous vein and sural nerve and the remaining ( *n* = 19; 26.39%) perforators were anterolateral. All these perforators were fasciocutaneous. The average size of the perforator at subfascial level from this segment of LCA was 1.34 mm ( Table 1 ). The knowledge gleaned from the cadaveric study was applied in our clinical study.

**Table 1:** Results of cadaveric study

Serial no.	Type of specimen	Total number of perforators from the retrofibular/retromalleolar segment of LCA	Perforator locations cranial to tip of lateral malleolus from the retrofibular/retromalleolar segment of LCA (cm)	Perforator location with respect to short saphenous vein and sural nerve	Average size of perforators at subfascial level (mm)
1	Fresh cadaver leg	3	3, 5, 6	All AL	1.2
2	Fresh cadaver leg	2	1, 7	All PM	1.15
3	Fresh cadaver leg	4	2, 4, 6, 7.5	All PM	1.25
4	Fresh cadaver leg	3	1.5, 3, 6	All PM	1.15
5	Amputation specimen	3	1, 3, 5	2 PM, 1 AL	1.3
6	Fresh cadaver leg	2	3, 6.5	All PM	1.15
7	Fresh cadaver leg	4	1, 3.5, 6, 7	3 PM , 1 AL	1.3
8	Fresh cadaver leg	2	1, 7	All PM	1.25
9	Fresh cadaver leg	3	2, 5, 7	All PM	1.5
10	Fresh cadaver leg	5	1, 1.5, 5, 6, 7	2 AL, 3 PM	1.75
11	Amputation specimen	2	1, 6	All AL	1.5
12	Fresh cadaver leg	3	2, 5, 6.5	All PM	1.15
13	Fresh cadaver leg	3	1, 3, 7	All PM	1.25
14	Fresh cadaver leg	3	3, 3.5, 7	All AL	1.5
15	Fresh cadaver leg	3	2.5, 6, 7	2 PM , 1 AL	1.35
16	Fresh cadaver leg	2	1, 7	All PM	1.15
17	Fresh cadaver leg	6	1, 3, 5, 5.5, 6, 7	3 PM , 3 AL	1.25
18	Fresh cadaver leg	3	2, 6, 7	2 PM , 1 AL	1.3
19	Fresh cadaver leg	4	1.5, 3, 6.5, 7	All PM	1.41
20	Fresh cadaver leg	5	2, 2.5, 6, 6.5, 7	3 PM , 2 AL	1.69
21	Fresh cadaver leg	2	3, 5.5	All PM	1.45
22	Fresh cadaver leg	5	2, 2.5, 3, 5, 6.5	All PM	1.44

Abbreviations: AL, anterolateral; LCA, lateral calcaneal artery; PM, posteromedial

## Clinical Study

### Materials and Methods

This prospective cohort study was conducted from January 2018 to May 2023 in 13 patients (10 males and 3 females) with posterior heel defects. Those with the extension of the wound into the plantar heel and inframalleolar regions were excluded from the study. Chronic ulcers of more than 2 months' duration were excluded from this study. Diabetic patients with peripheral vascular disease and chronic smokers were also excluded from this study. The study included posttraumatic posterior heel defects exposing tendoachilles insertion, with or without tendoachilles cut injury and subsequently reconstructed with LCA perforator flaps.

### Surgical Technique

Proximal to the defect, the perforators were located in the sulcus between the tendoachilles and peroneus longus using a 10-MHz handheld Doppler. Under the tourniquet control, the debridement was performed. The nondelineating exploratory incision was placed either through the previous scar or just anterior to the tendoachilles(since most of the perforators were posteromedial) as part of the V-Y advancement flap or the propeller flap. The dissection was performed over the tenosynovium of the tendoachilles to locate the LCA that was usually found deep to the sural nerve and the small saphenous vein. The course of the perforators was delineated in relation to the sural nerve and small saphenous vein. Similarly, the delineating incision was completed in the case of the V-Y advancement flaps. In the case of the perforator propeller flap, the nondelineating incisions were placed to locate the single best perforator. The single best perforator must be large in size and pulsatile, accompanied by venae comitantes. The classical biogeometry of the perforator propeller flap was incorporated in designing the flap, once the location of the single best perforator was determined and the delineating incision was completed. In the case of the V-Y advancement flap, one or two large perforators were included. In the case of the classical bilobed perforator propeller flap, the sural nerve and small saphenous vein were dissected away from the single best perforator and were preserved. The periperforator dissection was carefully done to provide a pedicle length of 0.75 to 1.50 cm. This allowed a gracious turn of the pedicle without kinking and twisting during the primary propeller movement of the flap. A trial inset was given to observe the perfusion at the business end of the flap after the usual anticlockwise rotation of the flap. After confirming the good perfusion, the remaining inset was given. In the V-Y advancement flap, preferably perforators were chosen on one side of the skeletonized and preserved sural nerve and small saphenous vein. The length of the flap usually will be two and a half to three times the advancing distance. The secondary defects of the propeller flaps were all skin-grafted in our series. Operated legs were immobilized in the posterior heel offloading Plaster of Paris slab. Windows were given in the dressing to monitor the flap. Globally offloading the operated foot, patients were ambulated under supervision after 24 hours using the walker. Graded compression bandages were applied from the third postoperative day onwards and sutures were removed on the twelfth day. In those cases where tendoachilles was repaired, plantar flexed posterior heel offloading aluminum splints were used from the third postoperative day onwards and maintained for an 8-week period. Partial weight bearing using the walker with passive stretching exercises was allowed for up to 8 weeks. Full weight bearing was allowed after 12 weeks. Once the sound healing of tendoachilles was confirmed by clinical examination (standing on the forefoot on the operated side), patients were allowed complete weight bearing. Patients were followed up at 6 monthly intervals. At the end of the follow-up period, they were assessed by two independent observers using the institutional combined aesthetic and functional outcome score ( Table 2 ).

**Table 2:** Institutional combined aesthetic and functional outcome score for posterior heel defects reconstruction

	Shoeability and ambulation of the reconstructed foot	4 - Easy and possible at all times with normal shoes and full range of mobility and ambulation
I.	-	3 - Difficult at times for fitting into shoes, painful range of movements perceived at times
	-	2 - Possible with only specially designed wide counter shoes and stiffness of the ankle and subtalar joints reducing the ambulatory capacity
	-	1 - Not possible even with wide shoes and severe painful restriction of movement
II.	Aesthetic deformity	4 - No aesthetic contour deformity; scars are nearly imperceptible
	-	3 - Mild bulge; scars are less perceptible
	-	2 - Large contour deformity; hypertrophic scar
	-	1 - Depression deformity of donor site; bulky flap
III.	Nerve injury and sensation at reconstructed site	4 - Sural nerve sensation intact; flap is having normal sensation
	-	3 - Sural nerve sensation intact with good protective sensation at reconstructed site
	-	2 - Persistent paresthesia > 3 months along sural nerve which is troublesome needing medical therapy
	-	1 - Anesthesia of sural nerve felt and reported spontaneously by the patient while sitting on folded legs with callosity developing over lateral malleolus and base of 5th metatarsal

**Table:** Interpretation of institutional score

Score	Interpretation
10–12	Good to excellent
8–9	Fair
≤ 7	Poor

### Case 1 Illustration

**Fig. 5:**
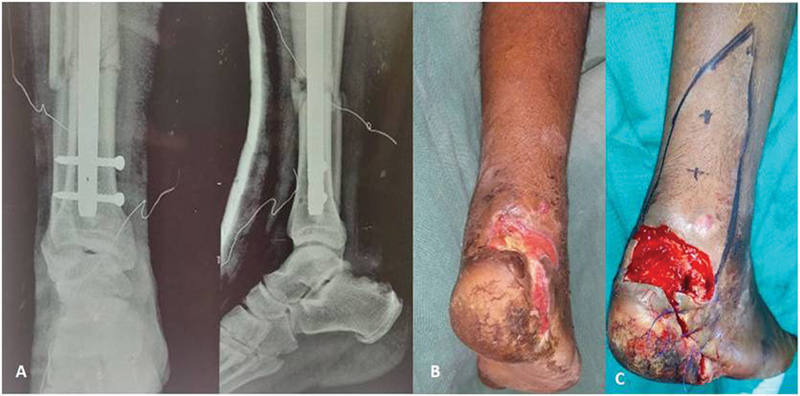
( **A** ) Showing nailing of the distal third both bone fracture of case 1. ( **B** ) Posterior heel defect of case 1. ( **C** ) Flap marking of case 1.

**Fig. 6:**
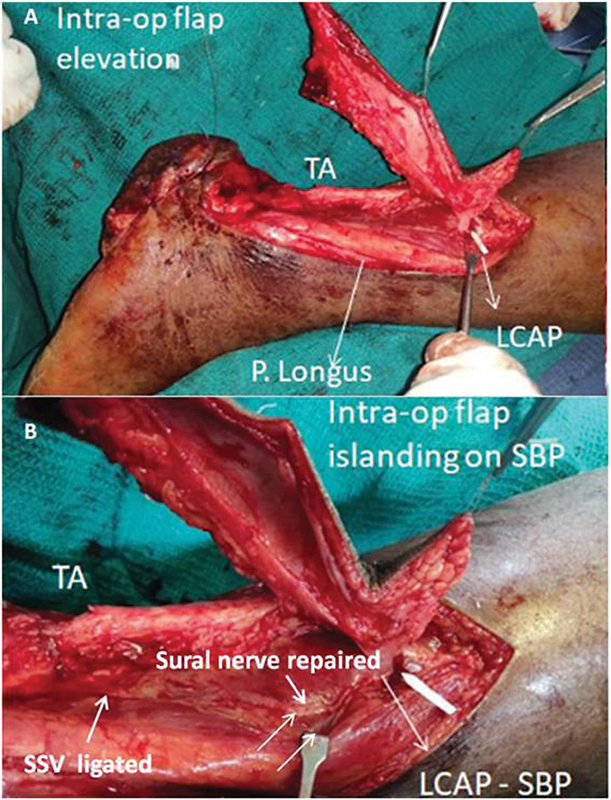
( **A** and **B** ) Intraoperative picture of case 1 showing the V-Y flap harvested on one large lateral calcaneal artery (LCA) perforator.

**Fig. 7:**
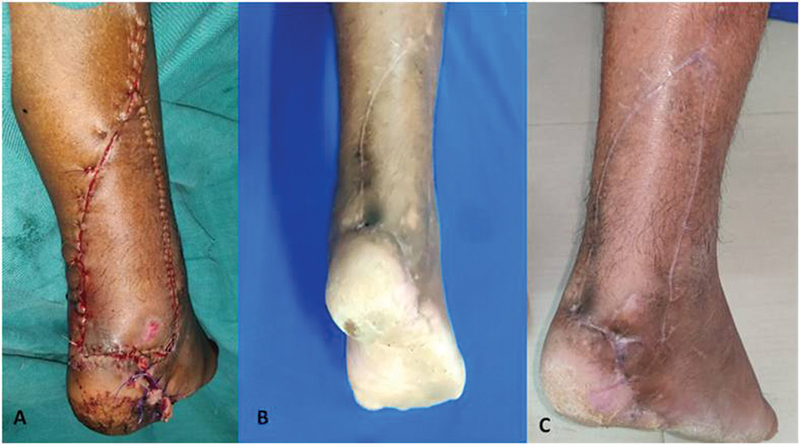
( **A** ) Immediate postop of case 1. ( **B** and **C** ) 18 months late postop of case 1.

A 34-year-old man ( Table 3 ) suffered a degloving injury to his right posterior heel in a traffic collision. He also sustained an open fracture to his right shaft of femur and a grade IIIB distal third leg both bone fractures on the same side. Skeletal fractures were stabilized with intramedullary nails ( Fig. 5A ). He was also diagnosed as diabetic at the time of admission. On exploration of the posterior heel degloving area, he had a complete laceration of tendoachilles. Krakow's repair was done for the tendoachilles laceration and primary closure was done after a thorough wash and debridement. On day 7, a second debridement of necrosed skin left behind a posterior heel defect measuring 3.75 × 4 cm ( Fig. 5B ) and loss of 30% of tendoachilles fibers which were removed as part of debridement. Two perforators 4 and 7 cm cranial to the tip of the lateral malleolus were marked by Doppler examination ( Fig. 5C ). V-Y advancement flap was marked. The length of the flap was taken triple the height of the defect with the advancing edge of the flap slightly wider than the width of the defect ( Fig. 5C ). The posteromedial incisions were made first and the flap was raised laterally in the suprafascial plane as far as the posterolateral peroneal septum. The single best pulsatile perforator from LCA was located 5.4 cm above the tip of the lateral malleolus ( Fig. 6A and B ). Note that 1.5 cm of periperforator dissection was done to prevent any compression by fascial strands and to facilitate V-Y advancement. The sural nerve was found to be cut and primarily repaired ( Fig. 6B ). Small saphenous vein was found thrombosed and was ligated at the distal end of the wound ( Fig. 6B ). The V-Y advancement and inset was given ( Fig. 7A ). Patient had uneventful healing of the wound ( Fig. 7B ). He had excellent institutional aesthetic and functional outcome score ( Table 2 ) of 12 at the end of 18 months follow-up period ( Fig. 7C ) ( Table 3 ). He had sound healing of tendoachilles ( Fig. 7C ).

**Table 3:** Demographic details of the patients

Serial no.	Age	Sex	Tendoachilles cut injury (TCI)/Exposure (TE)	Flap design	Defect size (cm)	Flap size	Perforator location cranial to tip of lateral malleolus from the retrofibular (RF), retromalleolar (RM) segment of LCA	Location of perforators from the retrofibular (RF) and retromalleolar (RM) segment of LCA	Perforator location with respect to short saphenous vein and sural nerve	Donor site closure	Follow-up (mo)	Institutional assessment score	Complications
1	48	M	TCI	V-Y advancement	4.2 × 3.0	9 × 4.5	6.4 (RF), 1.3 (RM)	RF, RM	All PM	Primary	17	12	None
2	52	F	TCI	V-Y advancement	3.6 × 2.4	8 × 3.5	6.8 (RF), 2 (RM)	RF, RM	All AL	Primary	19	10	None
3	29	M	TE	Classical bilobed propeller	5.5 × 4	10 × 5	6.4 (RF)	RF	All PM	STSG	12	10	Hypertrophied scar
4	32	F	TE	V-Y advancement	4.5 × 3.0	11 × 3.75	6.2 (RF), 3.4 (RM)	RF, RM	All PM	Primary	11	11	None
5	40	M	TCI	V-Y advancement	3.0 × 1.8	7.5× 3.5	6.8 (RF), 2.1 (RM)	RF, RM	All PM	Primary	10	12	None
6	43	M	TE	V-Y advancement	3.4 × 2.6	8 × 3.5	7.0 (RF), 2.8 (RM)	RF, RM	All PM	Primary	13	12	None
7	34	M	TCI	V-Y advancement	3.75 × 4	11 × 4.5	5.4 (RF)	RF	All AL	Primary	16	12	Temporary paraesthesia
8	38	M	TCI	V-Y advancement	4.0 × 3.2	10 × 5.5	6.2 (RF), 3.0 (RM)	RF, RM	All PM	Primary	17	12	None
9	33	M	TCI	Classical bilobed propeller	4.6 × 3	10.5 × 5	3.8 (RF)	RF	All PM	STSG	12	12	None
10	38	M	TCI	Classical bilobed Propeller	3.0 × 3.5	10.5 × 4.5	4.2 (RF)	RF	All PM	STSG	12	11	Superficial epidermolysis
11	32	F	TE	V-Y advancement	3.2 × 1.8	7.5× 4.5	5.5 (RF), 2.5 (RM)	RF, RM	All PM	Primary	19	10	Superficial Infection
12	28	M	TCI	V-Y advancement	3.8 × 2.0	8 × 4	4.5 (RF), 3.2 (RM)	RF, RM	All AL	Primary	15	11	None
13	45	M	TCI	V-Y advancement	5.0 × 4.6	9.2× 5.5	4.8 (RF), 2.5 (RM)	RF, RM	All PM	Primary	16	12	None

Abbreviations: AL, anterolateral; F, female; LCA, lateral calcaneal artery; M, male; PM, posteromedial; RF, retrofibular; RM, retromalleolar; STSG, split-thickness skin graft; TCI, tendoachilles cut injury; TE, tendoachilles exposed.

### Case 2 Illustration

**Fig. 8:**
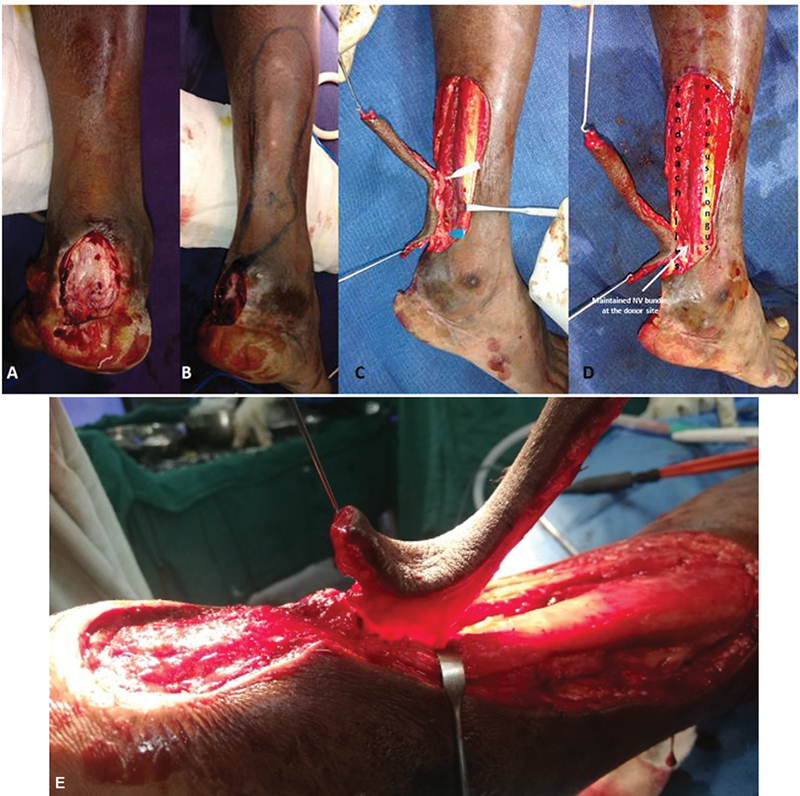
( **A** ) Posterior heel defect of case 2 following debridement. ( **B** ) Perforator propeller flap marking of case 2. ( **C** ) Case 2 perforator propeller flap on two posteromedial perforators from the lateral calcaneal artery (LCA). ( **D** ) Case 2 perforator propeller flap harvested on single best posteromedial perforator with maintenance of small saphenous vein and sural nerve at the donor site. ( **E** ) Case 2 showing islanded LCA propeller flap on a single best perforator.

**Fig. 9:**
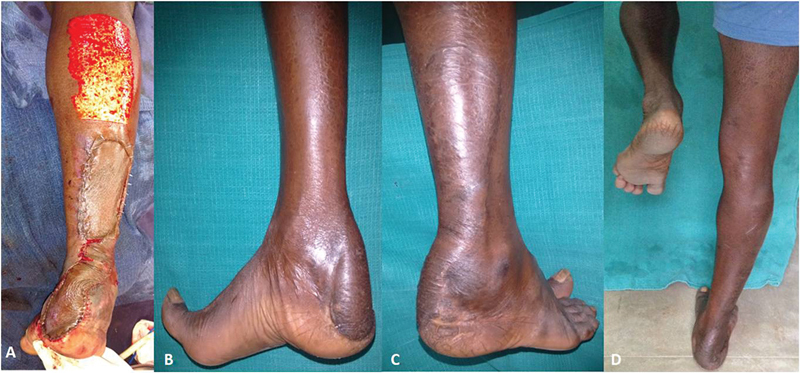
( **A** ) Case 2 immediate postop after flap inset. ( **B** and **C** ) 18 months late postop picture of case 2. ( **D** ) Late postop picture of case 2 showing the integrity of tendoachilles.

A 33-year-old fisherman with a history of closet injury sustained a complete cut of the right tendoachilles. The patient had primary repair of tendoachilles somewhere else and presented late to our clinic with necrotic soft tissue over the tendoachilles insertion with no overt invasive infection. The wound debridement was done resulting in the posterior heel soft tissue defect measuring 4.6 × 3cm ( Fig. 8A ) ( Table 3 ). A classical bilobed perforator propeller flap was planned after mapping of the perforators based on LCA ( Fig. 8B ). By placing the nondelineating posteromedial incision, two perforators were initially located ( Fig. 8C ) and then it was isolated on the single best perforator ( Fig. 8D ). Here again, small saphenous vein, sural nerve, and even the LCA were preserved at the donor site ( Fig. 8D ). The whole flap was islanded on a single best perforator ( Fig. 8 E ). The final inset was given by propelling the flap in the clockwise direction on a single best perforator ( Figs. 8E and 9A ). Patient was followed up for 12 months. He had a well-settled wound with complete healing of tendoachilles ( Fig. 9B and C ).At the end of the follow-up, he also had an excellent institutional aesthetic and functional outcome score of 12 ( Tables 2 and 3 ).

### Case 3 Illustration

**Fig. 10:**
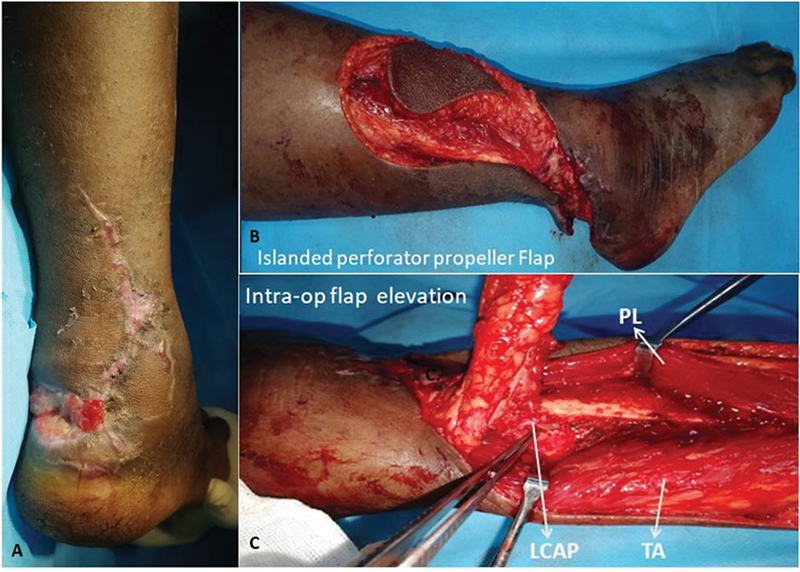
( **A** ) Case 3 preop posterior heel defect. ( **B** and **C** ) Case 3 perforator propeller flap harvested on a single best posteromedial perforator.

**Fig. 11:**
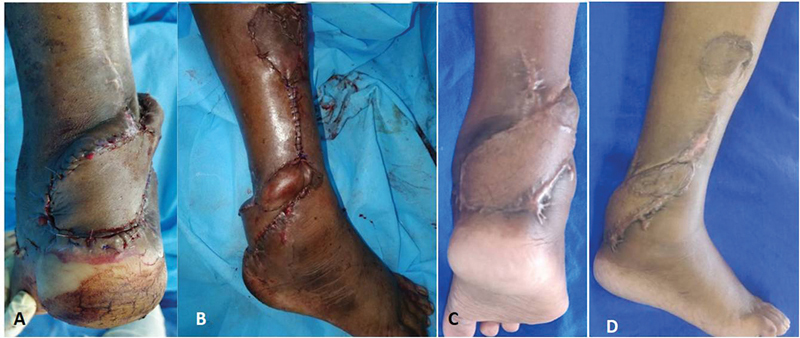
( **A** and **B** ) Case 3 immediate postop with flap inset. ( **C** and **D** ) Late postop of case 3.

**Fig. 12:**
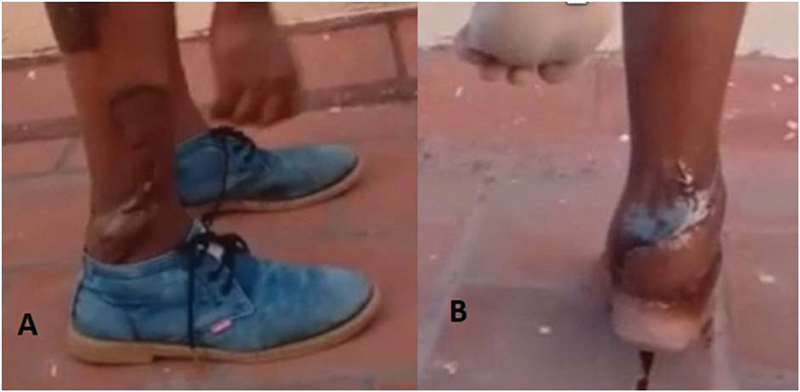
( **A** ) Shoeability of case 3. ( **B** ) Late postop picture of case 3 showing the integrity of repaired healed tendoachilles.

A 38-year-old male marketing professional sustained a degloving injury in a road traffic accident with partial tendoachilles cut injury. No fractures were present. Tendoachilles was repaired. On the seventh postoperative day, there was wound dehiscence with exposure of tendoachilles insertion ( Fig. 10A ). After the debridement, there was a soft tissue defect measuring 3.0 × 3.5 cm. A perforator propeller flap was marked with an anterior incision through the previous scar ( Fig. 10A ). A perforator propeller flap was harvested based on the single best perforator maintaining the neurovascular bundle at the sulcus between peroneus longus and the tendoachilles ( Fig. 10B and C ). He had superficial epidermolysis of the business end of the flap ( Table 3 ) which healed uneventfully ( Fig. 11B ). At the end of the follow-up, he had an institutional aesthetic and functional outcome score of 11 ( Tables 2 and 3 ) ( Fig. 11A–D ). He had sound healing of tendoachilles and well-settled flap facilitating shoeability ( Fig. 12A and B ).

## Results

The results of our study are given in Table 4 . In all our cases, LCA, small saphenous vein, and sural nerve were spared and maintained at the donor site. In one case, there was a cut injury of the sural nerve which was repaired intraoperatively. The rest of them did not have any paraesthesia/anesthesia in the cutaneous distribution of the sural nerve. Four patients had complications, but all of them were noncritical. There is a hundred percent survival rate of the flap in this study. The other three noncritical complications were superficial epidermolysis, hypertrophic scar, and superficial infection. All of them were treated conservatively. The maximum follow-up period in our series was 19 months and the minimum follow-up period in our series was 10 months. All the patients in our series have good to excellent scores as judged by two independent observers using our institutional aesthetic and functional outcome score ( Table 2 ). Note that 69.2% of patients had tendoachilles repair in our series ( *n* = 9). All of them had Krakow's repair (100%) done by a single surgeon. At the end of the follow-up period, all the patients were capable of standing on the toes/forefoot of the operated side without support, suggestive of sound healing of the tendoachilles (100%) ( *p* = 0.025). All of them were rendered shoeable with normal street shoes/footwear (100%) ( *p* = 0.025).

**Table 4:** Results of the clinical study

Factors	Results
Age range	28–52 y
Average age	37.84 y
Largest soft tissue defect	23 cm ^2^
Smallest soft tissue defect	5.4 cm ^2^
Average size of the soft tissue defect	12.34 cm ^2^
Largest size of the V-Y flap	55 cm ^2^
Smallest size of the V-Y flap	26.25 cm ^2^
Average size of the V-Y flap	38.48 cm ^2^
Largest size of the perforator propeller flap	52.25 cm ^2^
Smallest size of the perforator propeller flap	47.25 cm ^2^
Average size of the perforator propeller flap	49.92 cm ^2^
Average number of perforators situated posteromedial to SSV and SN	1.3 ( *n* = 10)
Average number of perforators situated anterolateral to SSV and SN	0.4 ( *n* = 3)
Location of the cranial most perforator from RF, RM segment of LCA (in cm cranial to the tip of lateral malleolus)	7.0
Location of the caudal most perforator from RF, RM segment of LCA (in cm cranial to the tip of lateral malleolus)	1.3
Percentage of complications	30.77% ( *n* = 4)
Average follow-up	14.53 mo
Average institutional and aesthetic functional assessment score	11.31

Abbreviations: LCA, lateral calcaneal artery; RF, retrofibular; RM, retromalleolar; SN, sural nerve; SSV, small saphenous vein.

## Discussion

There are certain anatomical idiosyncrasies associated with the posterior heel region. The skin in the posterior heel region is thin, pliable, and loosely attached. There is a paucity of loose expendable skin in this region. Any injury in this region, thus easily exposes the underlying tendoachilles attachment and the posterior aspect of the calcaneum. In addition, this region has high density innervation with plenty of sensory receptors and proprioceptive receptors. The continuous sensory and proprioceptive feedback from this region is necessary for normal gait and adept kinesiology of talocrural and subtalar joints. 13 Additionally, this area needs to tuck in snugly into the shoe or footwear counter. Any deformity in this area is not well tolerated because it makes shoeability difficult. So, the reconstruction of this region envisages the following: (1) The flap must be thin and pliable. 2. The flap should redrape well over the repaired or exposed tendoachilles reestablishing the concavity on either side of the tendoachilles seamlessly merging into the convexity of the heel region. Thereby, reestablishing the contour highlight of the posterior heel region. (3) The flap must be sensate to reinforce sensory and motor integration at the ankle region. (4) Reconstruction must facilitate shoeability. (5) The flap should have robust vascularity to cull the infection and contamination, that is common in the wounds of this region. (6) The flap should also promote extrinsic healing of the repaired tendoachilles. (7) The flap donor site morbidity must be minimal or negligible. All of the aforementioned optimal reconstructive needs are met by our proposed LCA perforator flap. As an islanded local perforator flap, it has color, thickness, and texture match. Therefore, upon healing it reestablishes the posterior heel contour highlights. The morbidity is essentially nonexistent at the donor site due to the sparing and preserving of the sural nerve, the small saphenous vein, and even the LCA. The sensate nature and supranormal homogenized blood supply 14 of the LCA perforator flap (later owing to the sympathectomy, flap staging, and eschewing the steal phenomenon of nonessential tissues) ensure that contaminated wounds heal without complications and make shoeability easier. The contour deformity of the donor site is minimal in LCA perforator flaps and it is also a microvascular surgery minus microvascular anastomosis. Wang et al 15 study on the LCA perforator flap is closely related to our work. But in their study, they raised a step ladder fasciocutaneous flap with small saphenous vein and sural nerve and had done only V-Y advancement. In our study, we have applied the knowledge gleaned from the anatomical study and spared and maintained small saphenous vein, sural nerve, and even the LCA at the donor site. When compared with their study, we have complete survival of all flaps with a noncritical complication rate. In addition, we have used a perforator propeller flap based on LCA and showed good survival. We have already explored perforator propeller flaps based on the terminal perforators of the peroneal artery. 1 But here in this study, we had concentrated only on perforators from the LCA. We have conducted the anatomical study to eschew controversial anatomy about LCA. What we found out by our dissection, there existed (as per Grabb and Argenta et al 11 and Sinnatamby 12 descriptions) a definitive retrofibular/retromalleolar segment anterior and medial to the flexor hallucis longus beefy portion and deviating peroneal tendons found with considerable number of perforators. Perforator-based flaps have been explored across almost all of the lower limbs except in the tendoachilles region. Our clinical study is the first of its kind introducing LCA perforator/propeller flap into the armamentarium of plastic surgery. The strength of our study is establishing the pattern and number of LCA perforators and its relation to the small saphenous vein and the sural nerve in an anatomical dissection. The ways and means of harvesting cutaneous island on the LCA perforators preserving and maintaining small saphenous vein, sural nerve, and even the LCA were established in our clinical study. The limitation is the small-size study. It may require a large-scale study to establish the veracity of the LCA perforator/propeller flap.

## Conclusion

Posterior heel defects are often difficult in their restoration because of their osseous or tendinous bed, poor area of vascularization, continuous movement, and high functional demands. Thus, LCA perforator/propeller flap may provide reliable cover for posterior heel defects. It provides good color, texture, and contour match reconstruction.
